# Psychological impact of COVID-19 on medical care workers in China

**DOI:** 10.1186/s40249-020-00724-0

**Published:** 2020-08-12

**Authors:** Ming-Yu Si, Xiao-You Su, Yu Jiang, Wen-Jun Wang, Xiao-Fen Gu, Li Ma, Jing Li, Shao-Kai Zhang, Ze-Fang Ren, Ran Ren, Yuan-Li Liu, You-Lin Qiao

**Affiliations:** 1 Department of Epidemiology and Biostatistics, School of Population Medicine and Public Health, Chinese Academy of Medical Sciences and Peking Union Medical College, Beijing, China; 2 School of Nursing, Jining Medical University, Jining, Shandong China; 3 Affiliated Tumor Hospital, Xinjiang Medical University, Urumqi, China; 4 Public Health School, Dalian Medical University, Dalian, China; 5 West China School of Public Health, Sichuan University/West China Forth Hospital, Sichuan University, Chengdu, China; 6 Henan Cancer Hospital, Affiliate Cancer Hospital of Zhengzhou University, Zhengzhou, China; 7 School of Public Health, Sun Yat-sen University, Guangzhou, China; 8 Global Health Research Center, Dalian Medical University, Dalian, China; 9 School of Health Management and Policy, Chinese Academy of Medical Sciences and Peking Union Medical College, Beijing, China; 10 Department of Cancer Epidemiology, National Cancer Center/National Clinical Research Center for Cancer/Cancer Hospital, Chinese Academy of Medical Sciences and Peking Union Medical College, 17 South Panjiayuan, Chaoyang District, Beijing, China

**Keywords:** COVID-19, Medical care worker, China, Psychological, Posttraumatic stress disorder, Depression, Anxiety, Stress

## Abstract

**Background:**

Medical care workers experienced unprecedented levels of workload and pressure since the outbreak of coronavirus disease 2019 (COVID-19). Little is known about its exact impact on medical care workers and related factors in China. This study aims to identify the psychological impact of COVID-19 on medical care workers in China.

**Methods:**

From February 23 to March 5, 2020, a cross-sectional survey was conducted among 863 medical care workers from seven provinces in China using standard questionnaires measuring adverse psychological outcomes including Impact of Event Scale-6 (IES-6), Depression, Anxiety and Stress Scale(DASS)and related psychosocial factors like perceived threat, social support and coping strategies. Exploratory Factor analysis was performed to identify the dimensions of perceived threat by study participants. Multivariate regression was used to examine the determinants of adverse psychological outcomes.

**Results:**

Posttraumatic stress (PTS) were prevalent in this sample of health care professionals, and 40.2% indicated positive screens for significant posttraumatic stress disorder symptoms. The proportion of having mild to extremely severe symptoms of depression, anxiety and stress were 13.6, 13.9 and 8.6%, respectively. Perceived threat and passive coping strategies were positively correlated to PTS and DASS scores, while perceived social support and active coping strategies were negatively correlated to DASS scores. Nurses were more likely to be anxious than others among medical care workers during the COVID-19 epidemic.

**Conclusions:**

Adverse psychological symptoms were prevalent among medical care workers in China during the COVID-19 epidemic. Screening for adverse psychological outcomes and developing corresponding preventive measures would be beneficial in decreasing negative psychological outcomes.

## Background

There have been 3 090 445 cases of coronavirus disease 2019 (COVID-19) and 217 769 death reported worldwide as of April 30, 2020 [1]. In China alone, there were reports of more than 84 373 COVID-19 cases with 4643 deaths [1]. After the rapid spread of the outbreak to many countries in the world, the World Health Organization (WHO) declared the COVID-19 outbreak as a pandemic on March 11, 2020. Public health intervention nationwide and quarantine had been implemented in most of the countries in the past months.

There is a wide consensus that the outbreak of an infectious disease is often linked with adverse psychological outcomes. Containment measures, including compulsory- or self-quarantine and social distancing, especially if protracted, may increase the risk of mental disorders, such as depression, anxiety, thought disorders and post traumatic stress (PTS) [2]. Compared with the general population, medical care workers are more likely to experience a wide range of negative psychological impact following an emergency or disaster. Severe emotional stress had been reported during or after the infectious diseases outbreak among medical care workers in previous studies, including the 2003 severe acute respiratory syndrome (SARS) epidemic [3], 2014 Ebola virus disease and 2015 Middle East respiratory syndrome (MERS) outbreak [4, 5].

It has been shown that the medical care workers experienced a high level of emotional stress, anxiety, depression and PTS during or even after the outbreak of the infectious diseases [6]. Acute stress disorder (ASD) has similar symptoms to post traumatic stress disorder (PTSD), is diagnosed three days to 1-month post trauma and is a good predictor of PTSD [7]. In a study among medical care workers in a Taiwan hospital during the outbreak of SARS, 5% suffered from an ASD [8]. Given the magnitude of pandemic of COVID-19 and the stress undergone by the medical care workers, adverse psychological outcomes are expected to occur among them, especially those on the front line. Up to now, little is known about the psychological impact of the COVID-19 pandemic on medical care workers in the most severely affected countries, including China.

Adverse psychological outcomes among medical care workers are usually determined by a variety of factors during an outbreak of infectious disease with high level of mortality, including uncertain quarantine duration, inadequate medical supplies, fears of infection, stigma and discrimination etc. [2, 9]. Meanwhile, the support they gained from others and the coping strategies they adopted during the event had been reported to be associated with their psychological status during the epidemic of infectious disease [5]. Less support and more negative coping strategies were proved to be common predictors of both acute and chronic PTS and other mental health problems [10, 11]. By understanding the psychological outcomes caused by an outbreak on medical care workers and studying the mechanism underneath, effective intervention and treatment can be developed and provided to this population, hence to improve their psychological wellbeing.

The present study aimed to investigate the presence of adverse psychological outcome, anxiety, depression and PTS, experienced by medical care workers during the COVID-19 outbreak and assess the associated factors, to better understand the psychological suffering of medical care workers and provide clues of developing intervention to alleviate the psychological stress of this population.

## Methods

### Sampling and data collection

Study participants were recruited from hospitals of seven geographical regions in China, located in north, south, east, central, northwest, northeast and southwest part of China. Clinical and administrative staff in these hospitals were invited to the study. They filled out an online self-administered structured questionnaire from February 23 to March 5, 2020. The questionnaires were disseminated and administered by a university staff in each of the geographical regions. Two pairs of questions were embedded in the survey questionnaire for quality control: (1) For basic information quality control, gender question was asked twice in the questionnaire in different places; (2) two reverse matching questions ‘Do you agree that influenza or pneumonia vaccine can prevent new COVID-19’ and ‘Do you agree that influenza or pneumonia vaccines cannot prevent COVID-19’ in the questionnaire. Only the questionnaires that pass the above triple quality control were included in the statistical analysis. Ethical approval for the survey was obtained from the Ethics Committee of Jining Medical University of Shandong Province on February 12, 2020.

### Measurements

Survey questions included demographic characteristics (i.e., age, gender, education, marital status and occupation), general health status, variables related to the COVID-19 (e.g. whether ever been quarantined, level of concern to the outbreak), perceived threat of COVID-19, perceived stress, anxiety, depression and PTS, perceived social support and coping strategies.

#### Impact of event Scale-6 (IES-6)

The impact of event Scale-6 (IES-6) was an abbreviated version of The Impact of Event Scale-revised (IES-R), and it includes three current symptom subscales of posttraumatic stress disorder (PTSD), including intrusion, hyper-arousal and avoidance. The six items of the IES-6 were proved correlated highly with the IES-R and the Post Traumatic Stress Disorder Check List – Civilian version (PCL), and was suggested to be a robust brief measure of posttraumatic stress reactions both in epidemiological studies and in clinical practice [12]. IES-R was validated among Chinese population and used for measuring the psychological impact after exposure to a crisis situation within one week of exposure [13]. In this study, the Cronbach’s alpha of IES-6 was 0.81.

#### Depression, anxiety and stress scale (DASS)

The Chinese brief version of the Depression, Anxiety, and Stress Scale (DASS-21) was used to measure psychological distress of the study participants. Study participants gave item ratings according to their experience over the past weeks on a 4-point Likert rating scale. It consists of three self-reported subscales (each with seven items), measuring depression, anxiety, and stress [14, 15]. The Chinese version of DASS had been validated among various Chinese population [16, 17]. The Chronbach’s alpha of the three subscales of DASS were 0.83, 0.80 and 0.82, and 0.92 for the total DASS in this study.

#### Perceived threat

Participants’ perceived threat by COVID-19 was measured by 8 items developed based on the earlier studies: ‘I am afraid of being infected by COVID-19’, ‘I’m anxious to be shifted to the ward for COVID-19 patients’, ‘I’m worried about being quarantined or isolated’, ‘My job puts me at a high risk of being infected by COVID-19’, ‘My close contacts are at high risk of being infected due to my job’, ‘My close friends and relatives are worried that I might transmit the virus to them’, ‘I’m distanced by others due to my job’, ‘I’m stigmatized by others due to my job’. For each individual item, the answer is on a 5-point Likert scale (‘strongly disagree’ to ‘strongly agree’), and a higher total score indicated a greater perceived threat by the COVID-19 outbreak. The Chronbach’s alpha of the 8-item perceived threat was 0.81.

#### The perceived social support scale (PSSS)

PSSS is a validated 12-item instrument, assessing perceived support obtained from family, friends, and significant others [18]. The scores range from 12 to 84, with a higher score indicating a higher level of perceived support. The Chinese version of the PSSS was validated and showed a good internal reliability (Cronbach’s alpha was 0.89) [19]. In this study, the Cronbach’s alpha value was 0.94.

#### Simplified coping style questionnaire

Coping style was assessed by the SCSQ, a 20-item scale with scores ranging from 0 to 3 on each item. SCSQ was developed based on the Ways of Coping questionnaire by Folkman and Lazarus [20, 21]. The SCSQ measures two coping styles: active coping (AC) and passive coping (PC), focusing on problem solving and emotional distress, respectively [22]. The scale has shown high internal consistency for both active coping styles (Cronbach’s α = 0.89) and passive coping styles (Cronbach’s α = 0.78). In this study, the Cronbach’s alpha coefficients for the two dimensions of SCSQ were 0.86 and 0.80, respectively.

### Statistical analysis

The internal consistency of the scales was assessed by using Cronbach’s alpha coefficients. Exploratory factor analysis, using principal component and varimax rotation methods, was performed Exploratory Factor analysis to uncover the underlying dimensions of the perceived threat items. The prevalence of psychological symptoms were derived according to the cut-off values suggested in previous studies. Descriptive statistics were calculated for sociodemographic characteristics and variables related to the COVID-19 outbreak. Linear regressions were used to calculate the univariate associations between sociodemographic characteristics, variables related to the COVID-19 outbreak, and psychological outcomes (the IES-6 score and the subscales of the DASS). Pearson correlation were performed to assess the associations between adverse psychological outcomes and the potential psychosocial factors of the perceived threat. Multiple linear regression was performed to explore adverse psychological health status and their potential factors by adjusting the variables significant in univariate analysis at *P ≤* 0.10. Statistical significance of all two-tailed tests was set at *P ≤* 0.05. The SPSS 22.0 (IBM SPSS Statistics, New York, United States) was used for the statistical analysis.

## Results

### Participants’ characteristics

Of the 1136 medical care workers we invited to the study, 863 completed the questionnaire survey (response rate = 76.0%). Table 1 presents the sample characteristics by adverse psychological outcomes. 29.3% were male, 77.3% were at age less than 40, 64.9% were currently married, 88.0% had a college or above educational background, 43.7% had a monthly income less than Chinese yuan 6000 (around United States dollar 850), 43.7% were doctors, 24.4% were nurses, 6.0% ever had chronic diseases, 7.4% were current tobacco users and 32.6% were current alcohol users. In addition, 25.6% had ever been quarantined or isolated during the outbreak, 16.8% were frontline medical workers, 74.0% were highly concerned about the epidemic. Respectively 95.0, 4.2 and 1.3% had confirmed cases in their living city, community and relatives and friends (Table 1 and Table 2).

**Table 1:** Association between demographics and adverse psychological outcomes of the COVID-19 outbreak (*n* = 863)

Variables	***n*** (%)	Impact of event		Depression		Anxiety		Stress	
		β	***P***	β	***P***	β	***P***	β	***P***
**Gender**									
Male	253 (29.3%)	-0.617	0.091	0.520	0.188	-0.135	0.714	0.169	0.730
Female	610 (70.7%)	Reference		Reference		Reference		Reference	
**Age (Years)**									
≤ 29	277 (32.1%)	0.233	0.754	-0.473	0.557	0.954	0.202	-1.089	0.274
30–39	390 (45.2%)	-0.410	0.572	-0.683	0.385	0·414	0.517	-0.968	0.320
40–49	145 (16.8%)	0.480	0.545	0.238	0.782	0.531	0.506	-0.008	0.994
≥ 50	51 (5.9%)	Reference		Reference		Reference		Reference	
**Marital status**									
Currently not married	303 (35.1%)	-0.230	0.508	-0.182	0.630	0.475	0.174	-0.397	0.395
Currently married	560 (64.9%)	Reference		Reference		Reference		Reference	
**Education**									
Technical secondary or below	104 (12.0%)	-0.198	0.722	0.170	0.778	0.431	0.441	0.084	0.910
College	466 (54.0%)	0.372	0.306	-0.134	0.733	0.450	0.219	-0.206	0.672
Advanced degree	293 (34.0%)	Reference		Reference		Reference		Reference	
**Monthly income (CNY)**									
< 6000	377 (43.7%)	-0.117	0.782	-0.158	0.728	0.442	0.296	0.164	0.771
6000–9999	277 (32.1%)	0.391	0.381	-0.445	0.358	0.066	0.883	-0.061	0.919
≥ 10 000	209 (24.2%)	Reference		Reference		Reference		Reference	
**Occupation**									
Doctor	377 (43.7%)	0.607	0.114	0.597	0.154	-0.046	0.905	0.398	0.443
Nurse	211 (24.4%)	1.520	0.001	0.699	0.148	1.283	0.004	0·957	0.110
Other health worker	275 (31.9%)	Reference		Reference		Reference		Reference	
**Ever had chronic disease(s)**									
Yes	52 (6.0%)	1.800	0.010	2.324	0.002	1.782	0.011	2.797	0.003
No	811 (94.0%)	Reference		Reference		Reference		Reference	
**Current tobacco user**									
Yes	64 (7.4%)	-1.426	0.024	-0.324	0.637	-0.733	0.250	-0.711	0.403
No	799 (92.6%)	Reference		Reference		Reference		Reference	
**Current alcohol user**									
Yes	281 (32.6%)	0.262	0.460	0.791	0.039	0.475	0.182	0.646	0.174
No	582 (67.4%)	Reference		Reference		Reference		Reference

**Table 2:** Association between variables related to the COVID-19 outbreak and adverse psychological outcomes (*n* = 863)

Variables	***n*** (%)	Impact of event		Depression		Anxiety		Stress	
		β	***P***	β	***P***	β	***P***	β	***P***
**Ever been quarantined or isolated**									
Yes	221 (25.6%)	0.407	0.285	0.776	0.059	0.308	0.421	0.610	0.232
No	642 (74.4%)	Reference		Reference		Reference		Reference	
**Duty during the epidemic**									
Front line workers	145 (16.8%)	0.156	0.725	0.012	0.980	0.817	0.067	0.938	0.115
Second line or others	718 (83.2%)	Reference		Reference		Reference		Reference	
**Levels of concern**									
High concern	639 (74.0%)	1.704	0.000	0.218	0.595	0.163	0.669	1.232	0.015
Less concern	224 (26.0%)	Reference		Reference		Reference		Reference	
**Confirmed cases in the living city**									
Yes	820 (95.0%)	0.983	0.197	-0.014	0.986	0.147	0.848	1.206	0.239
No or not sure	43 (5.0%)	Reference		Reference		Reference		Reference	
**Confirmed cases in the living community**									
Yes	36 (4.2%)	0.249	0.764	2.082	0.020	2.854	0.001	2.542	0.022
No or not sure	827 (95.8%)	Reference		Reference		Reference		Reference	
**Confirmed cases among relatives and friends**									
Yes	11 (1.3%)	3.045	0.039	5.265	0.001	3.563	0.017	7.808	0.000
No	852 (98.7%)	Reference		Reference		Reference		Reference	

### Factor loadings of perceived threat items and their psychometric properties

EFA on the 8 items of perceived threat yielded 3 factors (explaining 76.9% of the total variance; KMO = 0.75), with satisfactory eigenvalue and factor loadings (eigenvalue > 1 and factor loading > 0.45) [23]. The three factors identified from the EFA assess respondents’ level of perceived threat with respect to stigmatization/distancing, fears of infection and high-risk job, with Chronbach’s alpha of 0.81, 0.78 and 0.79 (Table 3). The overall score of perceived threat and the extracted factors were calculated by summing up all item scores and were proved all significantly correlated with the scores of IES-6 and three subscales of DASS (Table 4).

**Table 3:** Factor loadings of perceived threat items among medical care workers (*n* = 863)

Perceived threat (Items)	Factor1	Factor 2	Factor 3
	Stigmatization /distancing	Fears of infection	High-risk job
Afraid of being infected by COVID-19	-0.057	0.704	0.390
Anxious to be shifted to the ward for COVID-19	0.213	0.848	0.057
Worried about being quarantined or isolated	0.138	0.860	0.122
My job puts me at a high risk of being infected	0.119	0.297	0.820
My close contacts are at high risk of being infected	0.182	0.090	0.888
Friends and relatives are worried to be infected by me	0.627	0.110	0.498
I’m distanced by others due to my job	0.916	0.142	0.113
I’m stigmatized by others due to my job	0.901	0.087	0.079
Eigenvalue	2.158	2.091	1.899
Cumulative % of variance explained	26.971	53.111	76.845
Cronbach’s alpha	0.812	0.780	0.793

Exploratory factor analysis, using principle component analysis for factor extraction (with varimax rotation)

Factor 1–3 addressed threat due to stigmatization/distancing, fears of infection and high-risk job

**Table 4:** Pearson correlation between perceived threat, perceived social support, coping style and adverse psychological outcomes (*n* = 863)

Variables	Impact of event		Depression		Anxiety		Stress	
	β	***P***	β	***P***	β	***P***	β	***P***
**Perceived threat**	0.342	0.000	0.284	0.000	0.234	0.000	0.353	0.000
**Stigmatization/distancing**	0.517	0.000	0.674	0.000	0.548	0.000	0.761	0.000
**Fears of infection**	0.455	0.000	0.339	0.000	0.260	0.000	0.377	0.000
**High-risk job**	0.666	0.000	0.560	0.000	0.529	0.000	0.772	0.000
**Perceived social support**	-0.003	0.856	-0.109	0.000	-0.106	0.000	-0.130	0.000
**Active coping (AC)**	0.063	0.048	-0.127	0.000	-0.134	0.000	-0.172	0.000
**Passive coping (PC)**	0.234	0.000	0.309	0.000	0.247	0.000	0.318	0.000

### Prevalence of psychological symptoms and perceived threat

**Fig. 1:**
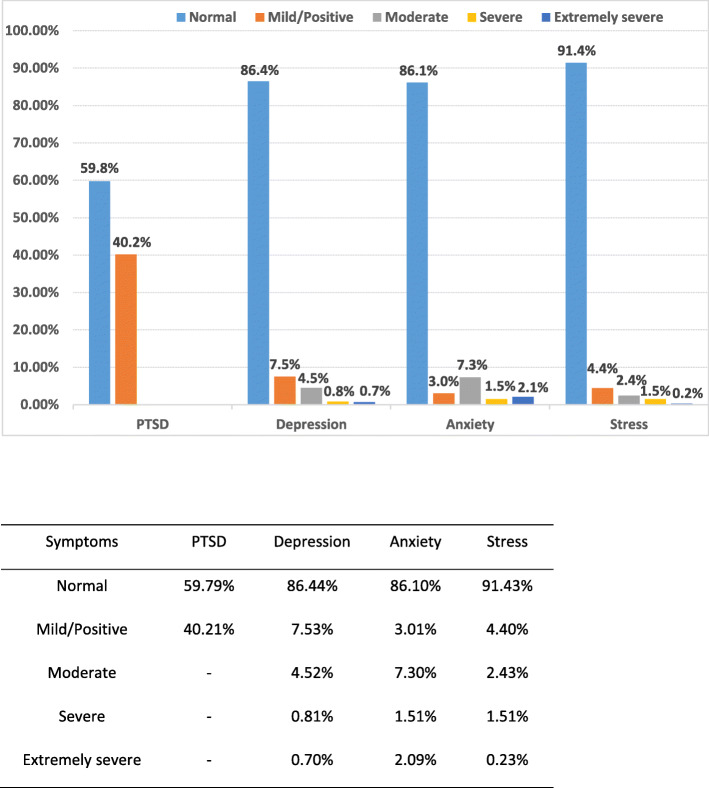
Percentage of participants with mild to extremely severe depression, anxiety and stress, and IES-6 scores more than the cut-off 10. PTSD: Post traumatic stress disorder; IES: Impact of event scale

**Fig. 2:**
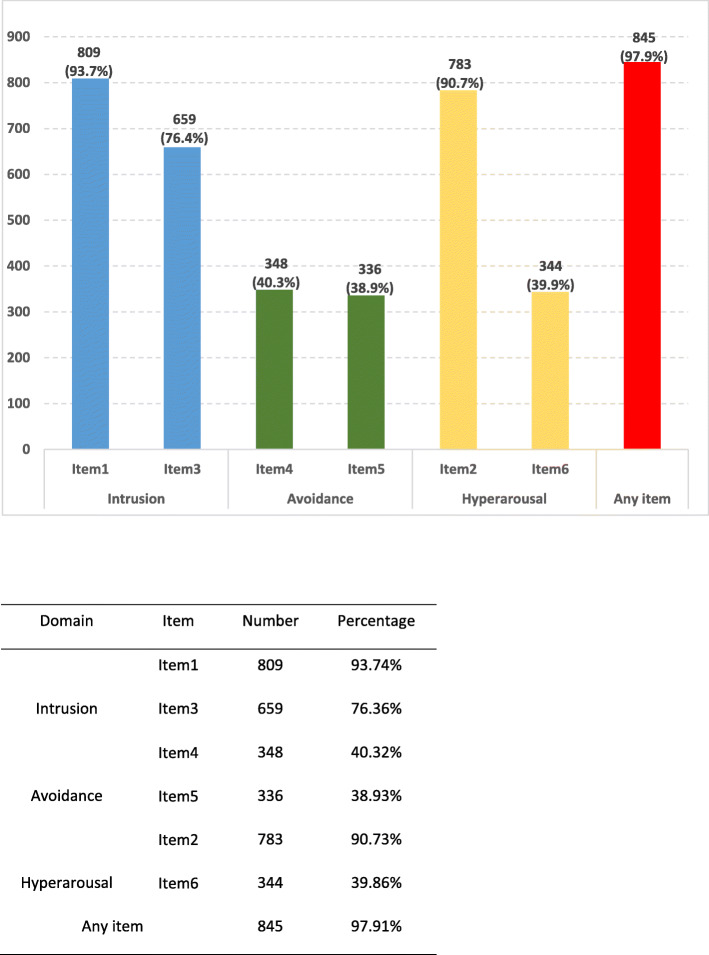
Percentage of participants with each IES-6 symptom and at least one IES-6 symptom. IES: Impact of event scale

IES-6 scale was used to measure the posttraumatic stress of COVID-19 outbreak, which revealed a sample mean score of 8.54 (standard deviation = 4.87) (data not tabulated). The IES-6 with a cutoff of 10 was used as a proximate measure of PTS, which is considered to have the best overall efficiency [12], and 347 (40.2%) were considered to meet the clinical concern of PTSD. 97.9% of the respondents had one or more PTS symptoms. The most severe PTS domain among the respondents was intrusion: ‘I thought about it when I did not mean to’ (93.7%) and ‘Other things kept making me think about it’ (76.4%), then the hyperarousal and avoidance domain (Fig. 1 and Fig. 2). DASS severity ratings of the respondents were presented in Fig. 1. The proportion of having mild to extremely severe symptoms of depression, anxiety and stress were 13.6, 13.9 and 8.6%, respectively.

**Fig. 3:**
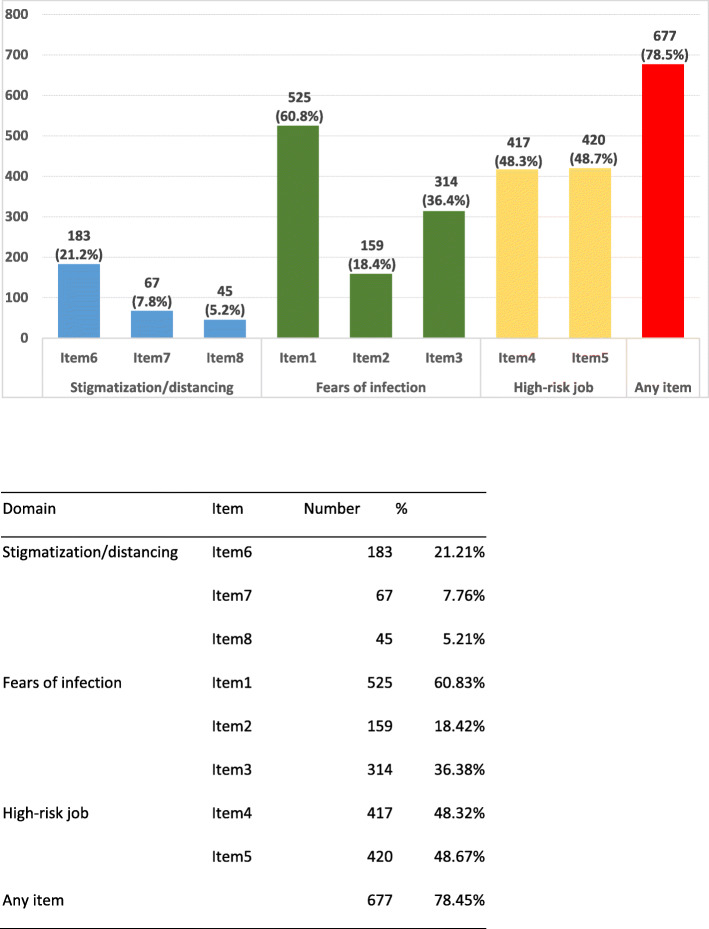
Percentage of participants with each perceived threat and at least one threat

In total, ‘Fears of infection’ and ‘Doing high-risk job’ were the mostly perceived threat by the participants. While 525 participants (60.8%) reported ‘I am afraid of being infected by COVID-19’, only 159 (18.4%) responded that ‘I’m anxious to be shifted to the ward for COVID-19 patients’. In addition, 420 (48.7%) agreed ‘My close contacts are at higher risk of being infected due to my job’, and 417 participants (48.3%) agreed ‘My job puts me at a high risk of being infected by COVID-19’. 78.5% of the participants reported have at least one out of the 8 perceived threat items (Fig. 3).

### Bivariate correlates of adverse psychological outcomes

Those who were nurses (β = 1.52, *P* = 0.001), ever had chronic diseases (β = 1.80, *P* = 0.010), had high concern to the outbreak (β = 1.70, *P* = 0.000), and had confirmed cases among their relatives and friends (β = 3.05, *P* = 0.039) were more likely to have PTS symptoms. Those who were current tobacco user were less likely to have PTS symptoms (β = -1.43, *P* = 0.024) (Table 1 and Table 2).

Those who ever had chronic diseases (β = 2.32, *P* = 0.002), who were current alcohol user (β = 0.79, *P* = 0.039), had confirmed cases in their living community (β = 2.08, *P* = 0.020) and had confirmed cases among their relatives and friends (β = 5.27, *P* = 0.001) were more likely to have depression symptoms. Those who were nurses (β = 1.28, *P* = 0.004), ever had chronic diseases (β = 1.78, *P* = 0.011) and had confirmed cases in their living community (β = 2.85, *P* = 0.001) and had confirmed cases among their relatives and friends (β = 3.56, *P* = 0.017) were more likely to have anxiety symptoms. Those who ever had chronic diseases (β = 2.80, *P* = 0.003), had high concern to the outbreak (β = 1.23, *P* = 0.015), had confirmed cases in their living community (β = 2.54, *P* = 0.022) and had confirmed cases among their relatives and friends (β = 7.81, *P* = 0.000) were more likely to have stress symptoms.

### Pearson correlation between adverse psychological symptoms and perceived threat, perceived social support and coping style

The IES-6 and DASS score were correlated with almost all proposed psychosocial variables including overall score and scores of the three domains of perceived threat, perceived social support, and two forms of coping strategies in anticipated directions. The adverse psychological symptoms were positively associated with perceived threat and passive coping strategies, while negatively associated with perceived social support and active coping strategies, except the correlation of IES-6 with perceived social support ((β = -0.00, *P* = 0.856) and with active coping (β = 0.06, *P* = 0.048) (Table 4).

### Multivariate correlates of psychological symptoms

Compare to other groups, nurses were more likely to have anxiety symptoms (β = 0.93, *P* = 0.026) (Table 5). Participants who were current tobacco user were less likely to have PTS symptoms (β = -1.55, *P* = 0.015). Level of concern to the outbreak was positively correlated with PTS (β = 1.49, *P* = 0.000) and stress symptoms (β = 1.22, *P* = 0.009). Those who had confirmed cases in their living community were more likely to be anxious (β = 1.82, *P* = 0.018), and who have confirmed cases among relatives and friends were more likely to have depression (β = 3.70, *P* = 0.011) and stress symptoms (β = 5.75, *P* = 0.002). Apart from the ‘Fears of infection’, other two dimension of perceived threat were positively associated with the PTS and DASS (value of β and *P* see Table 5), those with more fears of infection were more likely to have PTS (β = 0.25, *P* = 0.000). Three dimensions of Perceived threat and passive coping strategies were positively related to both PTS (β = 0.28, 0.25, 0.29 and 0.31, *P* = 0.000) and DASS symptoms (value of β and *P* see Table 5). Perceived social support and active coping were negatively related to DASS symptoms (value of β and *P* see Table 5). Those adopted passive coping strategies were more likely to have PTS and DASS.

**Table 5:** Results of multiple linear regression analysis on IES-6 and DASS (*n* = 863)

Variables	Impact of event		Depression		Anxiety		Stress	
	β	***P***	β	***P***	β	***P***	β	***P***
**Gender**								
Male	-0.140	0.721	-		-		-	
Female	Reference							
**Occupation**								
Doctor	-0.206	0.565	-		-0.473	0.187	-	
Nurse	0.549	0.201			0.930	0.026		
Other health worker	Reference				Reference			
**Ever had chronic disease(s)**								
Yes	1.174	0.068	1.198	0.080	1.067	0.095	1.357	0.111
No	Reference		Reference		Reference		Reference	
**Current tobacco user**								
Yes	-1.549	0.015	-		-		-	
No	Reference							
**Current alcohol user**								
Yes	-		0.344	0.323	-		-	
No			Reference					
**Ever been quarantined or isolated**								
Yes	-		0.588	0.111	-		-	
No			Reference					
**Levels of concern**								
Highly concerned	1.488	0.000	-		-		1.224	0.009
Less concerned	Reference						Reference	
**Confirmed cases in the living community**								
Yes	-		0.639	0.436	1.822	0.018	0.666	0.515
No or not sure			Reference		Reference		Reference	
**Confirmed cases among relatives and friends**								
Yes	2.071	0.125	3.701	0.011	1.792	0.190	5.747	0.002
No	Reference		Reference		Reference		Reference	
**Perceived threat**								
**Stigmatization/distancing**	0.282	0.000	0.416	0.000	0.306	0.000	0.433	0.000
**Fears of infection**	0.251	0.000	0.099	0.116	0.044	0.448	0.052	0.495
**High-risk job**	0.285	0.001	0.231	0.010	0.272	0.001	0.428	0.000
**Perceived social support**	-		-0.064	0.000	-0.072	0.000	-0.083	0.000
**Active coping (AC)**	0.031	0.303	-0.106	0.003	-0.106	0.001	-0.152	0.001
**Passive coping (PC)**	0.172	0.000	0.274	0.000	0.220	0.000	0.286	0.000

# Impact of event: R-Squared (*R*^2^) = 0.193, Adjusted R-Squared (AR^2^) = 0.182, *P* = 0.000; Depression: *R*^2^ = 0.217, AR^2^ = 0.206, *P* = 0.000; Anxiety: *R*^2^ = 0.199, AR^2^ = 0.189, *P* = 0.000; Stress: *R*^2^ = 0.200, AR^2^ = 0.191, *P* = 0.000

## Discussion

The results of our study revealed a high prevalence of PTS among Chinese medical care workers during the outbreak of COVID-19 virus. Nearly half of the participants suffered from PTS meeting clinical cut-off of PTSD and 97.9% experienced at least one PTSD symptoms, which was much higher than in other population in the same study (34.0 and 94.0% respectively among university students, data not tabulated). The rate was also out of the range of 10–27% probable and clinical PTSD diagnosis reported in Ebola epidemic during 2014–2016 in general population [9] and in SARS outbreak among medical care workers in 2003 [24]. Compare to the PTS symptoms, the prevalence of depression (13.6%) measured by DASS-21 were lower, but still significantly higher than the all age prevalence rate of 3.2% in Chinese population in recent decades [25].

Compared to the results of a multinational study on psychological impact of COVID-19 outbreak among health care professionals using the similar measurements, the proportion of PTSD symptoms, depression, anxiety, and stress were relatively higher in this study than in Singapore and in India, especially the prevalence of PTSD symptoms [26]. This difference may be explained from following aspects: Firstly, our study was conducted between February 23 to March 5, 2020, which lasted only two weeks at the beginning of the outbreak, while Chew’s study was carried out between February 19 to April 17, 2020, which lasted almost two months. In February 2020, China was the most affected country in the world by the COVID-19. Lack of knowledge and insufficient psychological coping strategies to the disease were very obvious under the overwhelming circumstances. Secondly, since the infrastructure and capability of the healthcare systems varies in different countries, the psychological reaction of health care professionals may differ during an outbreak of an infectious disease. However, another study among Chinese medical care workers also revealed a prevalence of depression, anxiety and stress symptoms, which was even higher than in this study, thus further indicated the factual psychological status in this population at the beginning of the COVID-19 outbreak [27].

Posttraumatic stress disorder (PTSD) is a common mental disorder manifesting through symptoms of intrusion, hyperarousal and avoidance following a traumatic event [28]. According to earlier studies, medical care workers are likely to develop adverse psychological problems, such as depression and post-traumatic stress as a result of their trauma experience [8, 29]. Under the circumstances of an infectious disease outbreak, the frontline medical care workers always have fears of being infected or infecting others, especially when they experience any physical symptoms related to the infection [2, 8, 30]. Meanwhile, with the lockdown of cities or even countries due to the COVID outbreak, the medical care workers became obviously the high-risk population to transmit the virus to whom have close contacts with them, and unsurprisingly, were under the situation of being stigmatized or distanced by others. In our study, EFA yielded three dimensions from the 8 perceive threat items, namely stigmatization/distancing, fears of infection and perceived high-risk of their job. These are highly concerned issues by medical care workers in the COVID-19 outbreak and in other similar epidemic, and were proved to be associated with adverse psychological outcomes in this study and previous others, especially PTSD symptoms [2, 6, 8, 9]. Therefore, apart from providing appropriate psychological counseling and accurate information targeting the stigmatization against the frontline health care workers to alleviate their perceived threat, a more supportive social environment and more friendly mass media would be helpful to medical care workers’ psychological health during an infectious disease outbreak. In addition, while the shortage on medical supplies among medical care workers is not very common in China in this pandemic, it is still worth considering in other countries and in future similar scenario, it may cause severe adverse psychological outcomes among them, even committing suicide.

Active coping strategies focusing on problem-solving can result in an improvement of person-environment relationship and thus lead to a positive emotional response [20]. Our data suggested strategies promoting active coping styles and providing sufficient social support may help to decrease the occurrence of adverse psychological symptoms like depression, anxiety and stress. This is consistent with the previous studies that active coping and social support were the most important buffering factors of negative psychological health among medical care workers [31, 32]. Our study also revealed passive coping strategies mainly focusing on the emotional distress were significantly related to a worse psychological health including PTS, depression, anxiety and stress symptoms. It can be explained that passive coping may lead to additional emotional exhaustion apart from the original stressful situation [22, 31]. The findings that active coping was not significantly related to PTSD symptoms demonstrated the importance of reducing passive coping strategies on PTSD among medical care workers in this extreme situation occurred very rarely in recent years and people have not prepared well to the pandemic both mentally and materially.

In this study, we found nurses were more likely to have anxiety symptoms compare to others. It can be easily interpreted that the nurses always contact patients with different illness, with various social-economic status, and directly access the patients’ blood sample, hence have the highest risk of being infected by the COVID-19 virus. Therefore, the occupational role of medical care workers should be considered in future outbreaks and the employers should encourage a supportive workplace to minimize the adverse psychological impact, and pay attention to the medical care worker with the most patient contact and most at risk [33]. Meanwhile, special attention should be taken to those with confirmed cases in their living community or among their acquaintances, they always have higher levels of concern to the epidemic, and hence prone to have adverse psychological symptoms.

Although current tobacco users were less likely to have PTS in our study, we could not agree that smoking is a proper way to alleviate PTS. A study recently published indicated that post-trauma anhedonia (PTA) is associated with increased substance use in a recently-traumatized population and PTA may be a mechanism through which substance use problems emerge in recently-traumatized individuals [34]. To those experienced PTS, caution should be taken to monitor their tobacco and substance use behaviour when taking care of their psychological health in the aftermath of a trauma event, since cigarettes and marijuana are very easy to be accessed in many countries for general people, not mention to the medical care workers.

Given the amount of stress experienced by the medical care workers during the pandemic, it is important to provide tailored mental health support to them, such as observing the trajectory changes of the post-pandemic mental health situation and establishing a nationwide psychological support group, to avoid the occurrence of widespread psychiatric disorders in this population. Otherwise, it would be a noticeable social and economic burden in the long run [35]. In addition, appropriate intervention measures should be adopted based on the psychological assessment in each stage of the pandemic, including timely counselling and screening, development of positive coping strategies, and create a more friendly social environment and mass media network. This would be applicable to similar epidemics in the future.

This study has several limitations. Since this is the first study of IES-6 utilized in Chinese medical care workers during the ongoing COVID-19 epidemic, the assessment of PTS symptoms using the cut-off value suggested in earlier studies does not necessarily accurate to suggest a clinical diagnosis of PTSD. Still, it can be a measure to identify those with significant PTS for further PTSD assessment and intervention. In addition, while we performed an EFA for extracting factors of perceived threat and proved an acceptable reliability and validity for further multivariate analysis, a more complicated validation analysis including confirmatory factor analysis (CFA) and others should be conducted in current sample and other study in the future. Furthermore, since this is a cross-sectional study conducted during the period when the COVID-19 outbreak was on a downward trend, caution should be made on causal relationships without further follow-up research. Despite the above limitations, the current study provided valuable information of the psychological reactions of medical care workers in China during the outbreak of COVID-19. Our results can provide references and guidance for future psychological interventions targeting this population.

In summary, adverse psychological symptoms were prevalent among medical care workers in China during the COVID-19 epidemic and a screening for PTS would be helpful to identify those might develop PTSD in the following months or years. The medical care workers experienced numerous threats including stigmatization, risk of being infected or infecting others, lack of necessary medical supplies and overwhelming workload. Lack of social support and maladaptive coping were important risk factors for occurrence of negative psychological outcomes among them. Preventive measures and mitigation strategies among medical care workers to prevent early traumatic stress reactions developing into chronic PTSD would be beneficial in decreasing adverse psychological outcomes [36].

## Conclusions

Adverse psychological symptoms were prevalent among medical care workers in China during the COVID-19 epidemic. Screening for adverse psychological outcomes and developing corresponding preventive measures would be beneficial in decreasing negative psychological outcomes of COVID-19 pandemic among medical care workers.

## Supplementary information

**Additional file 1.**

## Data Availability

The original data generated from this study and the analyzed results will be available from the corresponding author upon reasonable request.

## Abbreviations

COVID-19: Coronavirus disease 2019
IES-6: Impact of Event Scale-6
IES-R: Impact of Event Scale-revised
DASS: Depression, Anxiety and Stress Scale
PTS: Posttraumatic stress
PTSD: Posttraumatic stress disorder
SARS: Severe acute respiratory syndrome
MERS: Middle East respiratory syndrome
ASD: Acute stress disorder
WHO: World Health Organization
PCL-C: Post Traumatic Stress Disorder Check List – Civilian version
PSSS: The perceived social support scale
SCSQ: Simplified Coping Style Questionnaire
AC: Active coping
PC: Passive coping
EFA: Exploratory factor analysis
CFA: Confirmatory factor analysis
