# Supplementary material for: Psychological impact of COVID-19 on medical care workers in China
Source: Infect Dis Poverty. 2020 Aug 12;9:113. doi: 10.1186/s40249-020-00724-0 (PMC7422468; doi:10.1186/s40249-020-00724-0)
Supplement: Supplementary file 1 — Additional file 1. [file 40249_2020_724_MOESM1_ESM.docx]

**医务人员对新型冠状病毒感染肺炎的认知及情绪反应调查**

一、如果您同意参加本次调查，请勾选“我同意参加本次调查”选项；如果您不同意，请勾选“不同意”选项，且您的利益不会受到任何影响：

○我同意参加本次调查 ○不同意（无效问卷，跳转至结束作答）

**二、基本信息**

| 1.您此前是否被工作单位或社区要求居家或定点隔离？  ○ 是 ○ 否 |
| --- |
| 1. 您是否感染新型冠状病毒肺炎？   ○确诊感染 ○疑似感染 ○否（跳转至5.您的性别） |
| 3.您的确诊感染或疑似感染日期：________年______月 |
| 4.您的确诊感染或疑似感染地点是：________省______市 |
| 5.您的性别：  ○男 ○女 |
| 6.出生日期：________年______月 |
| 7.您的民族：  ○汉族 ○回族 ○藏族 ○维吾尔族 ○壮族 ○蒙古族 ○其他（请注明） |
| 8.您当前所在城市：_______省_______市 |
| 9.您的户口类型：  ○城镇 ○农村 |
| 10.您的婚姻状况：  ○未婚 ○在婚 ○婚内分居 ○离异 ○丧偶 |
| 11.您的受教育程度是  ○小学及以下 ○初中 ○高中/中专/技校/职高 ○大专 ○本科 ○研究生（包括硕士和博士研究生） |
| 12.近1年内您的月平均收入是：  ○无收入 ○小于2000元 ○2000元-3999元 ○4000元-5999元 ○6000元-7999元  ○8000元-9999元 ○≥1万元 ○≥1.5万 ○≥2万 ○≥4万 ○≥6万 ○≥8万 ○≥10万 |
| 13.您的执业类型：  ○医生 ○护士 ○防疫 ○其他卫生技术人员 |
| 14.您是否有以下慢性病史：（多选题）  ○ 心脑血管疾病（包括高血压、脑卒中和冠心病） ○ 癌症 ○糖尿病  ○慢性呼吸系统疾病 ○其他慢性病（请注明） ○无 |
| 15.目前您的身体健康状况如何？  很差 非常好  ① ② ③ ④ ⑤ ⑥ ⑦ |
| 16.您平时抽烟吗？（量表题）  ① ② ③ ④ ⑤ ⑥ ⑦ |
| 17.您平时饮酒吗？（量表题）  不喝 喝的很多  ① ② ③ ④ ⑤ ⑥ ⑦ |

**三、疫情相关信息**

| 1.您在此次新型冠状病毒肺炎疫情的防控中作为：  ○疫点一线医务人员 ○疫点二线医务人员 ○非疫点医务人员 |
| --- |
| 2.您对此次新型冠状病毒疫情的关注程度？  ○ 完全不关注 ○ 有所关注 ○ 比较关注 ○ 高度关注 |
| 3.截至目前，您当前所在的城市是否有确诊病例？  ○ 有 ○ 没有 ○ 不知道/不确定 |
| 4.截至目前，您当前所在的小区或村庄是否有确诊病例？  ○ 有 ○ 没有 ○ 不知道/不确定 |
| 5.据您目前所知，在此次疫情中，您的亲属和朋友中有没有人不幸感染新型冠状病毒的？  ○有 ○ 没有 |
| 6.这种说法您是否赞成：流感或肺炎疫苗能够预防新型冠状病毒肺炎。  ○是 ○ 否 |

**四、社会支持** 以下12个句子，每一个句子后面各有7个答案。请您根据自己平时的实际情况在每句后面选择一个答案。——领悟社会支持量表（Perceived social support scale,PSSS）

|  | 极不同意（1） | 很不同意（2） | 稍不同意（3） | 中立（4） | 稍同意（5） | 很同意（6） | 极同意（7） |
| --- | --- | --- | --- | --- | --- | --- | --- |
| 1.遇到问题时有些人（领导、亲友、同事）会出现在我的身边。 | □ | □ | □ | □ | □ | □ | □ |
| 2.我能够与有些人（领导、亲友、同事）共享快乐和忧伤。 | □ | □ | □ | □ | □ | □ | □ |
| 3.我的家庭能够切实具体的给我帮助。 | □ | □ | □ | □ | □ | □ | □ |
| 4.在需要时，我能从家庭获得感情上的帮助。 | □ | □ | □ | □ | □ | □ | □ |
| 5.有困难时有些人（领导、亲友、同事）是安慰我的真正源泉。 | □ | □ | □ | □ | □ | □ | □ |
| 6.我的朋友们能真正帮助我。 | □ | □ | □ | □ | □ | □ | □ |
| 7.在发生困难时，我可以依靠我的朋友们。 | □ | □ | □ | □ | □ | □ | □ |
| 8.我能与我的自己的家庭谈论我的难题。 | □ | □ | □ | □ | □ | □ | □ |
| 9.我的朋友们能够与我分享快乐和忧伤。 | □ | □ | □ | □ | □ | □ | □ |
| 10.我的生活中有些人（领导、亲友、同事）关心我的感情。 | □ | □ | □ | □ | □ | □ | □ |
| 11.我的家庭能心甘情愿协助我做出各种决定。 | □ | □ | □ | □ | □ | □ | □ |
| 12.我能与朋友们讨论自己的难题。 | □ | □ | □ | □ | □ | □ | □ |

**五、简易应对方式问卷（Simplified Coping Style Questionnaire, SCSQ）**  以下列出的是你在日常生活中经受挫折打击或遇到困难时可能采取的态度和做法，请在最适合您本人情况的选项打“√”

|  | 未采取（0） | 偶尔采取（1） | 有时采取（2） | 经常采取（3） |
| --- | --- | --- | --- | --- |
| 1.通过工作学习或一些其他活动解脱。 | □ | □ | □ | □ |
| 2.与人交谈，倾诉内心烦恼。 | □ | □ | □ | □ |
| 3.尽量看到事物好的一面。 | □ | □ | □ | □ |
| 4.改变自己的想法，重新发现生活中什么重要。 | □ | □ | □ | □ |
| 5.不把问题看得太严重。 | □ | □ | □ | □ |
| 6.坚持自己的立场，为自己想得到的斗争。 | □ | □ | □ | □ |
| 7.找到集中不同的解决问题的方法。 | □ | □ | □ | □ |
| 8.我向亲戚朋友或同学寻求建议。 | □ | □ | □ | □ |
| 9.改变原来一些做法或自己的一些问题。 | □ | □ | □ | □ |
| 10.借鉴他人处理类似困难情景的方法。 | □ | □ | □ | □ |
| 11.寻求业余爱好，积极参加问题活动。 | □ | □ | □ | □ |
| 12.尽量克制自己的失望、悔恨、悲伤和愤怒。 | □ | □ | □ | □ |
| 13.试图休息或休假，暂时把问题（烦恼）抛开。 | □ | □ | □ | □ |
| 14.通过吸烟、喝酒、服药和暴饮暴食来解除烦恼。 | □ | □ | □ | □ |
| 15.认为时间会改变现状，唯一要做的便是等待。 | □ | □ | □ | □ |
| 16.试图忘记整个事情。 | □ | □ | □ | □ |
| 17.依靠别人解决问题。 | □ | □ | □ | □ |
| 18.接受现实，因为没有其他办法。 | □ | □ | □ | □ |
| 19.幻想可能会发生某种契机改变现状。 | □ | □ | □ | □ |
| 20.自己安慰自己。 | □ | □ | □ | □ |

**六、认知题** 对于以下几种关于新型冠状病毒肺炎的说法，您持哪种态度？（请在选项上打“√”）

|  | 强烈反对  （1） | 反对  （2） | 不确定  （3） | 赞成  （4） | 十分赞成  （5） |
| --- | --- | --- | --- | --- | --- |
| 1我害怕感染到新冠病毒 | □ | □ | □ | □ | □ |
| 2担心自己被轮转到“新冠肺炎”病房 | □ | □ | □ | □ | □ |
| 3担心自己被隔离/活动受到限制 | □ | □ | □ | □ | □ |
| 4我的工作使我感染到新冠病毒的风险很高 | □ | □ | □ | □ | □ |
| 5因为我的工作，和我密切接触的人被感染新冠病毒的风险高 | □ | □ | □ | □ | □ |
| 6我关系密切的亲友都担心我会把新冠病毒传染给他们 | □ | □ | □ | □ | □ |
| 7由于自己的工作感到被家人或朋友疏远 | □ | □ | □ | □ | □ |
| 8感到自己被歧视 | □ | □ | □ | □ | □ |

**七、请评价以下陈述在多大程度上适合描述近两周的您？（**请你根据自己在新型冠状病毒肺炎流行期间的行为与感受，对照下面的每一条描述，选择最适当的答案。**）**

|  | 没有（0） | 轻微（1） | 有时（2） | 经常（3） | 总是（4） |
| --- | --- | --- | --- | --- | --- |
| ①我不由自主的会想到新冠肺炎这件事。 | □ | □ | □ | □ | □ |
| ②我变得警觉（戒备） | □ | □ | □ | □ | □ |
|  |  |  |  |  |  |
| ③其他的事也能让我联想起新冠肺炎疫情。 | □ | □ | □ | □ | □ |
| 基本信息质控题：如果您是男性请点击1，女性请点击5（与性别题比较，若匹配则质量较好） | 1 | 2 | 3 | 4 | 5 |
| ④我觉得我想了太多有关新冠肺炎的事情，我不知道怎样去处理 | □ | □ | □ | □ | □ |
| ⑤我试图不去想新冠肺炎这件事 | □ | □ | □ | □ | □ |
| ⑥我感觉自己难以集中注意力 | □ | □ | □ | □ | □ |

**八、请评价以下陈述在多大程度上适合描述近一周的你？——（Depression-Anxiety-Stress Scale 21,DASS-21)抑郁-焦虑压力量表**

|  | 不符合(0) | 有时符合(1) | 常常符合(2) | 总是符合(3) |
| --- | --- | --- | --- | --- |
| 1.我觉得很难让自己安静下来。 | □ | □ | □ | □ |
| 2.我感到口干舌燥。 | □ | □ | □ | □ |
| 3.我好像一点都没有感觉到任何愉快、舒畅。 | □ | □ | □ | □ |
| 4.我感到呼吸困难（气喘或透不过气来）。 | □ | □ | □ | □ |
| 5.我感到很难主动去开始工作。 | □ | □ | □ | □ |
| 6.我对事情往往做出过敏反应。 | □ | □ | □ | □ |
| 7.我感到颤抖（例如，手抖）。 | □ | □ | □ | □ |
| 8.我觉得自己消耗了很多精力。 | □ | □ | □ | □ |
| 9.我担心一些可能让自己恐慌或出丑的场合。 | □ | □ | □ | □ |
| 10.我觉得自己对不久的将来没有什么可期盼的。 | □ | □ | □ | □ |
| 11.我感到忐忑不安。 | □ | □ | □ | □ |
| 12.我感到很难放松自己。 | □ | □ | □ | □ |
| 13.我感到忧郁沮丧。 | □ | □ | □ | □ |
| 14.我无法容忍任何阻碍我继续工作的事情。 | □ | □ | □ | □ |
| 15.我感到快要崩溃了。 | □ | □ | □ | □ |
| 16.我对任何事情都不能产生热情。 | □ | □ | □ | □ |
| 17.我觉得自己不怎么配做人。 | □ | □ | □ | □ |
| 18.我发觉自己很容易被触怒。 | □ | □ | □ | □ |
| 19.即使在没有明显的体力活动时，我也感到心律不正常。 | □ | □ | □ | □ |
| 20.我无缘无故地感到害怕。 | □ | □ | □ | □ |
| 21.我感到生命毫无意义。 | □ | □ | □ | □ |

逆向匹配质控题

您是否赞成：流感或肺炎疫苗并不能预防新型冠状病毒肺炎。（与第三部分第6题形成逆向匹配）

○是 ○ 否
